# Strategies for supervising people with mental illnesses on probation caseloads: results from a nationwide study

**DOI:** 10.1186/s40352-023-00241-w

**Published:** 2023-10-12

**Authors:** Tonya B. Van Deinse, Mariah Cowell Mercier, Allison K. Waters, Mackensie Disbennett, Gary S. Cuddeback, Tracy Velázquez, Andrea Murray Lichtman, Faye Taxman

**Affiliations:** 1 School of Social Work, University of North Carolina at Chapel Hill, 325 Pittsboro Street, CB#3550, Chapel Hill, NC 27599 USA; 2 Utah Criminal Justice Center, University of Utah, 395 S 1500 E, Salt Lake City, UT 84112 USA; 3 School of Social Work, Virginia Commonwealth University, Academic Learning Commons, 3rd Floor, 1000 Floyd Avenue, P.O. Box 842027, Richmond, VA 23284 USA; 4 Safety & Justice Research, The Pew Charitable Trusts, 2005 Market Street, Suite 1700, Philadelphia, PA 19103 USA; 5 Schar School of Policy and Government, George Mason University, 3351 Fairfax Drive Van Metre Hall, Arlington, VA 22201 USA

**Keywords:** Probation, Mental illness, Prevalence, Screening, Supervision strategies

## Abstract

Probation officers are tasked with supervising the largest number of people living with mental illnesses in the criminal legal system, with an estimated 16–27% of individuals on probation identified as having a mental health condition. While academic research has recently focused on building the evidence base around the prototypical model of specialty mental health probation, less focus has been directed to the individual components of specialized mental health caseloads and other strategies agencies use to supervise people with mental illnesses. More specific information about these strategies would benefit probation agencies looking to implement or enhance supervision protocols for people with mental illnesses. This article describes the results from a nationwide study examining (1) probation agencies’ mental health screening and identification methods; (2) characteristics of mental health caseloads, including eligibility criteria, officer selection, required training, and interfacing with service providers; and (3) other strategies agencies use to supervise people with mental illnesses beyond mental health caseloads. Strategies for identifying mental illnesses varied, with most agencies using risk needs assessments, self-report items asked during the intake process, or information from pre-sentencing reports. Less than a third of respondents reported using screening and assessment tools specific to mental health or having a system that tracks or “flags” mental illnesses. Results also showed wide variation in mental health training requirements for probation officers, as well as variation in the strategies used for supervising people with mental illnesses (e.g., mental health caseloads, embedded mental health services within probation, modified cognitive behavioral interventions). The wide variation in implementation of supervision strategies presents (1) an opportunity for agencies to select from a variety of strategies and tailor them to fit the needs of their local context and (2) a challenge in building the evidence base for a single strategy or set of strategies.

On any given day, probation accounts for the largest percentage of people across the criminal legal system, with over 3 million people on probation caseloads nationwide (Kaeble, 2023). An estimated 16–27% of individuals on probation are identified as having a mental health condition; consequently, probation officers are tasked with supervising up to approximately 810,000 individuals with mental illnesses—more than any other sector in the criminal legal system (Crilly et al., 2009; Ditton, 1999; Kaeble, 2023). Individuals on probation who have mental illnesses face complex and interrelated challenges that increase barriers to accessing services, many of which create difficulties related to supervision compliance; these include housing instability, substance use, unemployment, trauma, comorbid physical health challenges, symptoms of mental illness, poor adherence to medication and treatment engagement, and eroding social support systems (Cuddeback et al., 2022; Garcia & Abukhadra, 2021; Longmate et al., 2021; Van Deinse et al., 2018; Yukhnenko et al., 2020).

Over the last 20 years, there has been a concerted effort to promote a rehabilitative approach to supervising people with mental illnesses by leaning away from a retributive orientation to one that focuses on addressing mental health-related challenges that can impact adherence to the terms of supervision. Although probation itself is not a tool or treatment for mental health recovery, a rehabilitative approach acknowledges that in order to improve probation outcomes, probation officers can facilitate resource connection, access to treatment, and address other barriers that inhibit successful completion of probation (Belenko et al., 2016; Taxman, 2008; Taxman & Caudy, 2015; Taxman et al., 2007; Viglione et al., 2015; Wooditch et al., 2014). Notably, there is a long history of shifting between retributive and rehabilitative orientations to probation over the last 50 years. For example, as a result of mass incarceration policies probation was enhanced with additional conditions to increase accountability. Rehabilitation was comingled within supervision to address some of the drivers of criminal conduct but rehabilitation was often secondary to the goals of compliance and accountability (Taxman & Breno, 2017).

The 2002 Criminal Justice/Mental Health Consensus Project was an illustration of the rehabilitative approach to supervision in which the Council of State Governments (CSG) encouraged jurisdictions to establish specialized strategies for supervising people with mental illnesses (Council of State Governments, 2002), such as reduced caseload sizes, enhanced mental health training for community supervision officers, and greater coordination and collaboration with treatment providers and other community resources. These strategies were further promoted by Skeem and colleagues (2006) in their seminal study, which codified the five elements of a prototypical model of specialty mental health probation: (a) specialized mental health caseloads consisting exclusively of people with mental illnesses, (b) reduced caseload sizes compared to general supervision, (c) ongoing mental health training for officers, (d) a problem-solving supervision orientation to address non-compliance and supervision challenges, and (e) collaboration and coordination with internal and external resources (Skeem & Louden, 2006). Since this study, a growing body of evidence has shown specialty mental health probation’s potential to improve mental health outcomes and criminal justice outcomes (Manchak et al., 2014; Skeem et al., 2017; Van Deinse et al., 2022; Wolff et al., 2014) and it was named a promising practice by Crime Solutions at the National Institute of Justice in 2022 (Office of Justice Programs, 2023).

With academic research focusing on building the evidence base around the prototypical model of specialty mental health probation, less focus has been directed to the individual components of specialized caseloads and other strategies that agencies use to supervise people with mental illnesses, or the efficacy of these components in improving supervision outcomes. More specific information about these strategies would benefit probation agencies looking to implement or enhance supervision protocols for people with mental illnesses. This article describes the results from a nationwide study examining (1) probation agencies’ mental health screening and identification methods; (2) characteristics of mental health caseloads, including eligibility criteria, officer selection, required training, and interfacing with service providers; and (3) other strategies agencies use to supervise people with mental illnesses beyond mental health caseloads. Where applicable, variation across rural and urban settings is described.

## Methods

### Design

Data for this cross-sectional study were collected between January 2021 and April 2022 and were obtained through survey methods and follow-up interviews with probation agency representatives from randomly selected counties across the U.S. Study methods were approved by the university’s institutional review board at the University of North Carolina at Chapel Hill.

### Sample

#### Survey respondents

Using a two-step process, the research team randomly selected 315 counties from the total population of 3,142 counties in the United States. In the first step of the sampling process, counties were stratified by state, then one county was randomly selected from each state to ensure representation of all states within the sample. The District of Columbia was selected with certainty. In the second step, 265 counties were randomly selected from the remaining 3,091 counties not selected in the first step for a total of 315 counties. The research team conducted outreach in each of the selected counties and requested contact information for agency representatives with substantial knowledge and experience with the agency’s strategies for supervising people with mental illnesses. Outreach efforts began in October 2020, and ended in July 2021, and were conducted in partnership with the American Probation and Parole Association. The research team obtained outreach information for all 315 counties selected. Of the 315 counties selected, representatives from 179 counties completed the survey (57% response rate). Notably, the study was conducted during the COVID-19 pandemic and agencies were tasked with implementing mandates to curb the spread of infection while also navigating internal workforce challenges related to COVID-19. These challenges likely contributed to a lower response rate; however, based on the research team’s analysis of non-response bias using data from the U.S. Census Bureau (U.S. Census Bureau, 2020), there were no statistically significant differences in county characteristics of responder counties versus non-responder counties (Table 1).

**Table 1:** County characteristics of survey respondents and non-respondents

	Survey respondents			Interview respondents		
	Total (*n* = 315)	Respondent (*n* = 179)	Non-respondent (*n* = 136)	Total (*n* = 179)	Completed interview (*n* = 26)	Did not complete interview (*n* = 153)
Rurality %(*n*)						
Completely or mostly rural	39.37 (124)	41.34 (74)	36.76 (50)	41.34 (74)	50.00 (13)	39.87 (61)
Mostly urban	60.63 (191)	58.66 (105)	63.24 (86)	58.66 (105)	50.00 (13)	60.13 (92)
County Population M(SD)	262,188.80 (505,800.20)	255,856.90 (392,431.60)	270,522.80 (625,996.90)	255,856.90 (392,431.60)	333,606.40 (483,061.20)	242,644.60 (375,174.30)
Unemployment M(SD)						
2019	3.83 (1.35)	3.79 (1.22)	3.89 (1.52)	3.79 (1.22)	4.04 (1.30)	3.75 (1.20)
2020	7.17 (2.15)	7.05 (1.99)	7.32 (2.35)	7.05 (1.99)	7.37 (1.70)	7.00 (2.03)
Median income M(SD)	62,430.92 (18,704.88)	6097.44 (17,858.99)	64,343.97 (19,666.12)	60,977.44 (17,858.99)	63,254.12 (21,527.05)	60,590.55 (17,211.71)
% Hispanic ethnicity M(SD)	10.86 (12.04)	10.37 (11.07)	11.49 (13.22)	10.37 (11.07)	9.51 (8.81)	10.52 (11.43)
Race (non-Hispanic) M(SD)						
White/Caucasian	75.34 (19.80)	75.60 (19.69)	74.99 (20.02)	75.60 (19.69)	73.99 (21.48)	75.87 (19.43)
Black/African American	9.81 (13.34)	10.23 (13.77)	9.25 (12.78)	10.23 (13.77)	13.26 (16.27)	9.71 (13.30)
American Indian and Alaska Native	1.82 (6.97)	2.12 (7.77)	1.43 (5.74)	2.12 (7.77)	1.45 (3.79)	2.24 (8.26)
Asian	3.10 (5.07)	3.02 (4.92)	3.21 (5.28)	3.02 (4.92)	3.31 (4.38)	2.97 (5.01)
Native Hawaiian and Other Pacific Islander	0.17 (0.90)	0.11 (0.24)	0.25 (1.34)	0.11 (0.24)	0.16 (0.36)	0.10 (0.21)
Gender M(SD)						
Male	51.48 (5.06)	51.52 (4.95)	51.41 (5.22)	51.52 (4.95)	51.51 (5.00)	51.52 (4.96)
Female	51.86 (4.87)	52.13 (5.05)	5.15 (4.61)	52.13 (5.05)	52.21 (5.52)	52.12 (4.99)

A majority of survey respondents were male (59%, *n* = 106), White (75%, *n* = 135), and had at least a 4-year college degree (91%, *n* = 162; Table 2). Survey respondents from urban counties were slightly older than respondents from rural counties (M = 48.43, SD = 8.26 vs. M = 45.63, SD = 9.04, respectively; *p* < 0.05) but were comparable on all other respondent characteristics. Overall, respondents had worked within community supervision for an average of 19 years (SD = 9.13), at their agencies for 18 years (SD = 9.18), and in their current positions for 8 years (SD = 7.90). Nearly half of the respondents (48%, *n* = 85) in the sample were managers or supervisors, 25% (*n* = 45) were community supervision officers, 22% (*n* = 40) were administrators or directors, one respondent was an office manager, and eight did not respond to this question. More than a quarter of respondents (26%, *n* = 46) had previously worked in mental health or substance use services.

**Table 2:** Survey and interview respondent characteristics

	Total (*n* = 179)	Rural (*n* = 74)	Urban (*n* = 105)	Completed interview (*n* = 26)	Did not complete interview (*n* = 153)
Gender %(n)					
Female	34.64 (62)	35.14 (26)	34.29 (36)	57.69 (15)	30.72 (47)
Male	59.22 (106)	56.76 (42)	60.95 (64)	42.31 (11)	62.09 (95)
Prefer not to answer	1.68 (3)	2.70 (2)	0.95 (1)	0 (0)	1.96 (3)
Missing	4.47 (8)	5.41(4)	3.81 (4)	0 (0)	5.23 (8)
Age (*n* = 165) M(SD)	47.28 (8.67)	45.63 (9.04)*	48.43 (8.26)	45.77 (9.83)	47.56 (8.45)
Latino/Latina/Latinx %(n)	3.35 (6)	2.70 (2)	3.81 (4)	0 (0)	3.92 (6)
Race %(n)					
American Indian or Alaska Native	1.68 (3)	1.35 (1)	1.90 (2)	0 (0)	1.96 (3)
Asian	0.56 (1)	0.00 (0)	0.95 (1)	0 (0)	0.65 (1)
Black or African American	12.85 (23)	13.51 (10)	12.38 (13)	11.54 (3)	13.07 (20)
White	75.42 (135)	72.97 (54)	77.14 (81)	88.46 (23)	73.20 (112)
Prefer not to say	4.47 (8)	6.76 (5)	2.86 (3)	0 (0)	5.23 (8)
Missing	5.03 (9)	5.41 (4)	4.76 (5)	0 (0)	5.88 (9)
Highest level of education %(n)					
High school diploma or GED	1.68 (3)	4.05 (3)	0.00 (0)	0 (0)	1.96 (3)
Some college	1.12 (2)	1.35 (1)	0.95 (1)	0 (0)	1.31 (2)
2-year degree	2.23 (4)	1.35 (1)	2.86 (3)	3.85 (1)	1.96 (3)
4-year degree	54.75 (98)	59.46 (44)	51.43 (54)	50.00 (13)	55.56 (85)
More than 4-year college degree	35.75 (64)	28.38 (21)	40.95 (43)	46.15 (12)	33.99 (52)
Missing	4.47 (8)	5.41 (4)	3.81 (4)	0 (0)	5.23 (8)
Field of concentration %(n)					
Criminal justice	47.49 (85)	54.05 (40)	42.86 (45)	57.69 (15)	45.75 (70)
Sociology	8.94 (16)	8.11 (6)	9.52 (10)	0 (0)	10.46 (16)
Psychology	10.61 (19)	10.81 (8)	10.48 (11)	19.23 (5)	9.15 (14)
Social work	3.35 (6)	0.00 (0)	5.71 (6)	7.69 (2)	2.61 (4)
Other^a^	22.35 (40)	16.22 (12)	26.67 (28)	15.38 (4)	23.53 (36)
Missing	7.26 (13)	10.81 (8)	4.76 (5)	0 (0)	8.50 (13)
Number of years in current position (*n* = 171) M(SD)	7.95 (7.90)	8.07 (8.39)	7.87 (7.59)	6.58 (5.43)	8.20 (8.26)
Number of years at agency (*n* = 171)	17.58 (9.18)	16.24 (8.87)	18.51 (9.32)	15.58 (9.18)	17.94 (9.17)
Number of years in community supervision (*n* = 171)	19.35 (9.13)	17.23 (9.02)*	20.81 (8.96)	18.08 (9.13)	19.57 (9.10)
Previously worked in mental health or substance use services %(n)					
Previously worked in mental health service	7.82 (14)	6.76 (5)	8.57 (9)	3.85 (1)	8.50 (13)
Previously worked in substance use services	2.79 (5)	1.35 (1)	3.81 (4)	0 (0)	3.27 (5)
Previously worked in both mental health and substance use services	15.08 (27)	12.16 (9)	17.14 (18)	15.38 (4)	15.03 (23)
Did not previously work in mental health or substance use services	69.83 (125)	74.32 (55)	66.67 (70)	80.77 (21)	67.97 (104)
Missing	4.47 (8)	5.41 (4)	3.81 (4)	0 (0)	5.23 (8)
Agency characteristics at the county level					
Number of people on probation (*n* = 172) M(SD)	1870.08 (3646.19)	380.68 (959.13)***	2968.31 (4432.97)	3443.42 (7080.48)*	1589.89 (2551.87)
Median	576.50	150	1500	709.50	500
Standard caseload size (*n* = 162)					
M (SD)	356.76 (1124.27)	113.13 (168.70)	537.52 (1453.74)	113.13 (168.70)*	537.52 (1453.74)
Median	80	75	85	86	80

^a^Examples of other fields include: business, communications, education, government, history, human resources, occupational therapy, public administration, political science

* *p* < .05

#### Interview participants

In addition to the web-based survey, the research team conducted interviews with a subsample of survey respondents to obtain additional details about agency strategies for supervising people with mental illnesses. Of the 179 survey participants, 85 (47%) expressed interest in participating in the interview and 26 completed interviews (31% response rate). On average, interviews lasted 35 min and were conducted via Zoom or telephone. Interviews were conducted over 8 weeks between April 21, 2021, and June 17, 2021. Exceptions to this timeframe were made for three interviews that were conducted by April 26, 2022, after final approval by a probation agency’s institutional review board. Low response rates for interview participation were likely impacted by ongoing challenges related to the COVID-19 pandemic. Non-response bias was examined using data from the U.S. Census Bureau (U.S. Census Bureau, 2020), and there were no statistically significant differences in the characteristics of counties represented in the interviews and those not represented (Table 1).

The average age of interview participants was 46 (SD = 9.83), the majority were White (88%, *n* = 23) and more than half were female (58%, *n* = 15). The vast majority had at least a four-year degree (96%, *n* = 25), most of whom had a degree in criminal justice (58%, *n* = 15). Interview participants had worked in their current position an average of 7 years (SD = 5.43), worked at their agency for 16 years (SD = 9.18), and had been working in community supervision for 18 years (SD = 9.36). More than half of the interview participants (58%, *n* = 15) were managers or supervisors, 27% (*n* = 7) were community supervision officers, 12% (*n* = 3) were administrators or directors, and one interview participant (4%) was an office manager.

#### Probation census in respondent counties

Of the 179 respondents, 59 (*n* = 105) represented urban counties and 41% (*n* = 74) represented rural counties. The average probation census across counties participating in the survey was 1,870 (SD = 3,646.19). Compared with rural counties, the probation census in urban counties was significantly larger (M = 380.68, SD = 959.13 vs. M = 2968.31, SD = 4432.97, respectively; *p* < 0.001; Table 2). Standard (i.e., non-specialty) caseload sizes varied widely and the average was 357 (SD = 1124.27) with a median caseload of 80 (Table 2). Average standard caseload sizes were significantly larger in urban counties (M = 537.52, SD = 1453.74) compared with rural counties (M = 113.13, SD = 168.70; *p* < 0.01). However, the median caseload size across rural counties was 75 compared to a median of 60 in urban counties.

### Measures

#### Web-based survey

The web-based survey was developed in collaboration with key stakeholders with (1) firsthand experience as a mental health probation officer, (2) administrative experience in community supervision, or (3) extensive experience in probation-related research. Additionally, the survey was reviewed by an external expert in the field of probation. To establish a common understanding of mental illness across respondents, the following definition was provided in the survey:

- For the purposes of this study, the term mental illness refers to either: (1) a mental illness, such as schizophrenia, bipolar disorder, depression, generalized anxiety disorder and/or post-traumatic stress disorder (PTSD), that has been diagnosed by a medical or mental health provider; (2) individual self-report of a diagnosis from a medical or mental health provider; or (3) a potentially undiagnosed mental illness that has been flagged using screening or assessment instruments that may be part of a probation department’s documentation or intake process. Although substance use disorder is considered a mental illness and often is presented alongside other psychiatric illnesses, within the context of this survey, the term “mental illness” does not refer to people whose only mental illness is a substance use disorder.

The survey consisted of 59 items and was organized into five sections: (1) general information about adult probation; (2) processes for identifying people on probation with mental illnesses; (3) information about the agency’s strategies for supervising people with mental illnesses (e.g., officer training, designated caseloads, etc.); (4) information about standard caseloads (i.e., non-specialty caseloads); and (5) respondent information and demographics. In Sect. 3 of the survey, respondents were asked to identify up to five mental health probation approaches or strategies used in their county. They were then asked to select one of those strategies named and then answer additional questions about the characteristics of the selected strategy.

#### Semi-structured interview guide

There were three sections of the semi-structured interview guide and this article focuses on Sect. 2: (1) challenges supervising people with mental illnesses, (2) implementation of the agency’s specialty mental health approach, and (3) COVID-19-related challenges and program adaptations. Results from Sect. 1 of the interview guide are reported elsewhere (Waters at al., 2023) and results from Sect. 3 have not yet been published.

### Data analysis

#### Quantitative analysis

To examine rural and urban variation, counties were categorized based on the proportion of their respective populations living in rural areas, according to the 2010 U.S. Census (U.S. Census Bureau, 2012). Counties considered “mostly rural” (i.e., 50%- 99% of the county population was living in rural areas) or “completely rural” (i.e., 100% of the county was living in rural areas) were labeled rural. Counties considered “mostly urban” (i.e., less than 50% of the county was living in rural areas) were labeled urban. Frequencies, counts, means, and standard deviations were used for univariate analyses. Chi-square tests, Fisher’s exact tests, independent sample t-tests, and Wilcoxon rank sum tests were used for bivariate analyses. Analyses were conducted in Stata 17 (Stata Statistical Software, 2021).

#### Qualitative analysis

To analyze participant interviews, the research team used general inductive coding methods and consensus coding (i.e., 100% agreement between coders). During the coding process, research team members reviewed a set of four interviews and developed an initial codebook. The team then compared and revised codebooks until final agreement was reached. The remaining interviews were divided between pairs of research team members who first independently coded their transcripts then compared codes. Each coding pair discussed all discrepancies until agreement was reached. Any codes unable to be resolved by the pair were adjudicated by the the larger coding group. Analyses were conducted in Dedoose (Dedoose, n.d.). General inductive coding (Thomas, 2006) was also used to analyze open-ended items from the survey or survey response options marked as “Other,” which included an option to input additional information.

Qualitative analyses were used to analyze Section 3 of the survey where respondents were asked to identify up to three strategies their agency implemented at the county level to supervise people with mental illnesses. Two research team members used general inductive coding methods to identify counties that reported having at least one mental health caseload as well as other types of supervision strategies. Coding to identify mental health caseloads and additional supervision strategies was based on (1) the descriptive name respondents gave to identify their mental health probation approach(es); (2) answers to questions about caseload size and designated caseloads; and (3) any open-ended text from this section of the survey that pertained to the identified supervision strategy. Two research team members compared coding results and discussed discrepancies until agreement was reached. The research team then summarized the quantitative survey results for those coded as mental health caseloads to describe caseload composition, required training, modification of sanctions, and other features of mental health caseloads. Qualitative analyses for this step were conducted in Microsoft Excel (Microsoft Corporation, 2018).

## Results

Results are organized into the following sections: (1) mental health screening and identification, (2) description of mental health caseloads in terms of size, composition, eligibility, officer training, service provider contact, and more; and (3) additional supervision strategies for people with mental illnesses. As applicable, both quantitative survey data and qualitative results from the follow-up interviews are integrated into their corresponding sections.

### Mental health screening and identification

#### Screening instruments and other sources of mental health information

Less than a third of respondents reported using standalone mental health screening tools (27%; *n* = 47), and 11% (*n* = 29) reported using mental health assessment tools to identify people on probation with mental illnesses. On the other hand, a majority of survey respondents reported using mental health-related questions on risk-needs assessment tools (72%, *n* = 126). Survey respondents named a number of risk assessment tools and other measures as part of their screening process (e.g., Correctional Offender Management Profiling for Alternative Sanctions, Level of Services Case Management Inventory [LS/CMI], Ohio Risk Assessment System; Andrews et al., 2004; Brennan et al., 2009; Latessa et al., 2010). These risk and need assessment instruments identified may not have mental health screening items or may not have been validated tools (Desmarais et al., 2018).

Additionally, more than two-thirds of respondents (67%, *n* = 117) used self-report items on agency intake forms and 63% (*n* = 109) used information on pre-sentencing investigative reports (Table 4) to identify people who may have a mental illness. Many agencies access multiple sources for mental health-related information. One interviewee indicated a person could be put on a mental health caseload via a judge’s order, results of a screening tool, or information in administrative prison records.

In addition to screening instruments, some agencies have data management systems that can record assessment and screening information. Just over a quarter of respondents (28%, *n* = 51) reported that their agencies used an electronic management system that had a mental health “flag.” These flags can indicate the need for further assessment or potentially placing a person on a specialized caseload. One interview participant indicated that people were placed on a mental health caseload either through a universally administered screening or through a diagnosis reported by a treatment provider or the individual themselves. Use of screening and assessment tools and electronic management systems with a mental health flag was comparable across rural and urban counties with no statistically significant differences (Table 3).

**Table 3:** Mental health screening and identification, availability of mental health caseloads

	Total (*n* = 179)	Rural (*n* = 74)	Urban (*n* = 105)
Screening tools %(*n*) (*n* = 174)			
Risk needs assessment	72.41 (126)	76.71 (56)	69.31 (70)
Mental health screening tool	27.01 (47)	27.40 (20)	26.73 (27)
Mental health assessment tool	10.92 (19)	10.96 (8)	10.89 (11)
Self-report on agency intake	67.24 (117)	65.75 (48)	68.32 (69)
Other self-report or disclosure	55.17 (96)	50.68 (37)	58.42 (59)
Pre-sentencing investigative report	62.64 (109)	54.79 (40)	68.32 (69)
Pre-trial assessment/report or court record	42.53 (74)	35.62 (26)	47.52 (48)
Other	25.29 (44)	21.92 (16)	27.72 (28)
Missing	2.79 (5)	1.35 (1)	3.81 (4)
Electronic management system has a mental health flag (*n* = 160) %(n)	28.49 (51)	28.38 (21)	28.57 (30)
Agency tracks mental illness	37.99 (68)	35.14 (26)	40.00 (42)
Prevalence of mental illness (*n* = 59) M(SD)	23.99 (18.78)	21.56 (18.00)	25.77 (19.41)
Prevalence based on estimate (*n* = 43)	24.63 (20.03)	20.37 (17.46)	28.00 (21.61)
Prevalence based on agency data (*n* = 11)	20.37 (16.30)	27.50 (23.98)	16.29 (10.10)
Prevalence based on other source (*n* = 5)	26.46 (13.89)	21.00 (19.80)	30.10 (11.85)
Mental health probation caseload (*n* = 179)***	27.37 (49)	10.81 (8)	39.05 (41)

***p < .001

#### Prevalence of mental illness among people on probation

Of the 179 counties, a little over a third (38%; *n* = 68) reported that they track the number of people on probation with a mental illness. Of the 68 respondents that track mental illness, 59 provided prevalence rates for an overall average of 24% (SD = 18.78) of people on probation. Prevalence rates based on estimates (*n* = 43), those based on agency data (*n* = 11), and those based on other sources (*n* = 5) were comparable, meaning there were no statistically significant differences between them. Additionally, prevalence rates were comparable across rural and urban counties with no statistically significant differences.

### Mental health caseloads: size, composition, eligibility, officer training, and sanctions

Of the 179 counties represented in the study, 27% (*n* = 49) had a mental health probation caseload and urban counties were significantly more likely to have a mental health caseload compared to rural counties (39%, *n* = 41 vs. 11%, *n* = 8, respectively; *p* < 0.000). In the section that follows, data describing mental health caseloads are based on 66 mental health caseloads identified by 49 counties. Where applicable, additional details from follow-up interviews or open-ended survey items pertaining to mental health caseloads are provided.

#### Caseload size and composition

Across the 66 mental health caseloads, the average caseload size was 43 (SD = 22.36) and 62% (*n* = 40) of caseloads were designated exclusively for people with mental illnesses (i.e., 100% of people on the caseload had a mental illness), 29% (*n* = 19) had mixed caseloads with a majority of people on the caseload diagnosed as having a mental illnesses, and 6% (*n* = 4) had mixed caseloads with up to half of people on the caseload diagnosed with a mental illness (Table 4).

**Table 4:** Mental health caseloads

	%(*n*)
Size and composition^1^	
Mean caseload size (*n* = 62)	42.84 (22.36)
Median	40
Percent of caseload designated for people with mental illnesses (*n* = 65)	
100% have a mental illness	61.54 (40)
75% to 99% have a mental illness	29.23 (19)
50% have a mental illness	6.15 (4)
< 50% have a mental illness	1.54 (1)
Unsure	1.54 (1)
Eligibility criteria (*n* = 49)	
Clinical diagnosis	91.84 (45)
Self-reported mental illness	44.90 (22)
Mental health flag on screening instrument	40.82 (20)
Other type of mental health eligibility^1^	32.65 (16)
Exclusionary offenses	
Excludes sex offenses	61.22 (30)
Excludes violent offenses	12.24 (6)
Excludes other offense types^2^	38.78 (19)
Approach allows any sentence length	93.88 (46)
Mental health training %(n)	
Type of training	
Mental Health First Aid	65.31 (32)
General risk-need-responsivity principles	63.27 (31)
Agency-developed mental health training	57.14 (28)
Mental health crisis de-escalation training	51.02 (25)
Crisis Intervention Team	40.82 (20)
Other type of training	20.41 (10)
No training required	8.16 (4)
Number of hours required	
Mean	25.14 (28.00)
Median	13.5
Frequency of mental health training booster sessions	
Annually	44.90 (22)
Booster sessions not required	28.57 (14)
Unsure	18.37 (9)
Other	8.16 (4)
Officer selection	
Recommendation from supervisor	63.27 (31)
Officer volunteer	46.94 (23)
Years of experience	44.90 (22)
Assessment of officer competency completed by supervisor	36.73 (18)
Education	32.65 (16)
Assessment of officer competency completed by people on the officer’s caseload	10.20 (5)
Assessment of officer competency based on officer self-assessment	8.16 (4)
No criteria for selection	14.29 (7)
Other	28.57 (14)
Reasons for contact with local service providers	
Referrals for service	97.96 (48)
Check on compliance/attendance	97.96 (48)
Seek guidance and resources for people on caseload	93.88 (46)
Problem-solving challenges related to people on caseload	91.84 (45)
Verification of medications	89.80 (44)
Verification of diagnosis	85.71 (42)
Host a case consultation or treatment team meeting	69.39 (34)
Request for medical records	61.22 (30)
Other reasons for contacting providers	10.20 (5)
No contact with providers	2.04 (1)
Sanctions and Modifications	
Degree of flexibility to modify sanctions for mental health caseloads (*n* = 49)	
Same flexibility	67.35 (33)
More flexibility	28.57 (14)
Less flexibility	4.08 (2)
Flexibility to seek modifications to probation conditions (*n* = 49) %(n)	
Same flexibility	79.59 (39)
More flexibility	20.41 (10)

^1^Some respondents reported more than one mental health caseload in their county. Data pertaining to size and composition are based on the 66 mental health caseloads identified in the sample

^2﻿^Examples of other types of charge exclusions: no exclusions, domestic violence or intimate partner charges, security risk group (e.g., gang)

In terms of counties’ (*n* = 49) eligibility criteria for mental health caseloads, 92% (*n* = 45) accepted individuals with a clinical diagnosis, 45% (*n* = 22) accepted a self-report of mental illness, and 41% (*n* = 20) accepted a mental health ‘flag’ or indicator on a screening instrument. In terms of counties’ exclusionary offenses for mental health caseloads, 61% (*n* = 30) reported excluding individuals with sex offenses and 12% (*n* = 6) excluded those with violent offenses. Lastly, the majority of counties with mental health caseloads (94%, *n* = 46) did not restrict eligibility based on probation sentence length.

Survey results pertaining to caseload size, composition, and eligibility were consistent with follow-up interviews during which participants described reduced caseloads sizes of around 40 people, variation in whether counties had mixed or designated mental health caseloads, and mandated criteria (e.g., specific diagnoses) or exclusions (e.g., such as people with intellectual or developmental disabilities). One participant described their agency’s mental health caseloads:

We have eight officers that are what we consider our mental health initiative caseloads, and those are grant-funded caseloads. Those officers focus mostly on that Axis 1 diagnosis…but also anyone that has a mental impairment that prohibits them from being able to just function at full capacity…So, we do have a lot of PTSD, and like I mentioned before, the generalized anxiety disorder.

Another participant described their mental health caseload unit and how their agency prioritizes caseload assignment:

We have 9 [probation officers] and about 350 clients or so. …We only take folks with psychotic disorder or bipolar 1… if we expanded the criteria to cover everything that you had mentioned as far as diagnostic stuff, we would more than triple the size of the unit. We are not able to do that. Folks who do not fit in those diagnostic categories of psychotic disorder or bipolar 1 generally go on a general caseload, and then the [probation officer] coordinates with a community-based mental health treatment provider.

#### Training for mental health officers

Across the 49 counties reporting details of their mental health caseloads, 65% (*n* = 32) required Mental Health First Aid, a training developed and disseminated by the National Council for Mental Wellbeing, 63% (*n* = 31) required general risk-need-responsivity principles training, 57% (*n* = 28) required an agency-developed mental health training, 51% (*n* = 25) required a mental health crisis de-escalation training, and 41% (*n* = 20) required Crisis Intervention Team (CIT) training. The average number of mental health training hours across the 49 counties reporting mental health caseloads was 25.14 (SD = 28.00) and the median was 13.5 h. In addition, 45% (*n* = 22) required annual booster sessions, and 29% (*n* = 14) reported that booster training sessions were not required (Table 5).

**Table 5:** Mental health training for standard (i.e., Non-mental Health) probation officers

	Total (*n* = 179)	Rural (*n* = 73)	Urban (*n* = 99)
Mental health training requirement for standard officers (*n* = 178) %(n)	35.75 (64)	44.59 (33)	29.52 (31)
Average number of mental health training hours among required (*n* = 64) M(SD)	8.09 (7.06)	7.53 (5.72)	8.68 (8.33)
Frequency of mental health training booster sessions for required training (*n* = 64) %(n)			
Annually	62.50 (40)	72.73 (24)	51.61 (16)
Every other year	6.25 (4)	9.09 (3)	3.23 (1)
Booster sessions not required	21.88 (14)	9.09 (3)	35.48 (11)
Other	9.38 (6)	9.09 (3)	9.68 (3)
Crisis Intervention Team (*n* = 179) %(n)	8.38 (15)	9.46 (7)	7.62 (8)
Other mental health crisis de-escalation training (*n* = 179) %(n)	46.93 (84)	50.00 (37)	44.76 (47)
Mental Health First Aid (*n* = 179) %(n)	28.49 (51)	33.78 (25)	24.76 (26)

Participants in the follow-up interviews provided additional details pertaining to officer training. Mental health training varied across agencies in terms of: who receives the training (e.g., all officers vs. only mental health probation officers); frequency (e.g., once, annually, ad hoc); voluntariness (i.e., required, voluntary, recommended); content (e.g., risk assessments, motivational interviewing, trauma, etc.); and who administers it (e.g., probation agency, outside treatment provider). While some agencies provide the same mental health training for all officers, others offer extra training and support for mental health officers (i.e., those supervising specialized mental health caseloads), with one participant saying that “they're going to institute an officer forum where officers can talk to other officers around the state, mental health officers, to just discuss cases and have just a safe spot for them.”

Officer training also varies in terms of frequency of course offerings. For instance, some agencies offer annual trainings to help officers “stay current”: “We do it yearly, and we change it a little bit each year. We try to see what is going on in the community, as well as in the country, and try to just make sure we're varying so that it's not the same for everybody.”

Other agencies offer one-time trainings, such as Mental Health First Aid. One respondent stated that “each probation officer has to keep up with doing training every year but they can kind of pick and choose which topics they do.” Training instruction and facilitation can also vary, including developing their own trainings, bringing in external trainers (e.g., academic partners or local university representatives, mental health agencies) with more expertise, and receiving internet-based training.

#### Officer selection

In terms of officer selection, 63% (*n* = 31) of counties reporting details of their mental health caseloads required a recommendation from the officer’s supervisor, 47% (*n* = 23) sought officers who volunteered to be a mental health officer, and 45% (*n* = 22) considered an officer’s years of experience. Additional consideration for officer selection for mental health caseloads included a supervisor’s assessment, education level of officer, assessment completed by people on the officer’s caseload, and a self-assessment.

#### Referral and coordination with service providers

Of the counties reporting details of their mental health caseloads, 98% (*n* = 48) contacted providers to make referrals for services and 98% (*n* = 48) contacted providers to check on compliance and attendance. In addition, mental health officers in counties engaged in more collaborative contacts with service providers. For instance, in 94% (*n* = 46) of counties reporting on their mental health caseloads, officers contacted service providers to seek guidance about people on their caseloads and in 92% (*n* = 45) of counties with mental health caseloads, officers contacted providers to problem-solve challenges related to people on their caseloads. Additional reasons for contacting service providers were to verify medications (90%, *n* = 44), verify a diagnosis (86%, *n* = 42), host a case consultation or treatment team meeting (69%, *n* = 34), and request medical records (61%, *n* = 30).

Consistent with the survey results, participants in the follow-up interviews described how the level of coordination and collaboration varied across agencies. First, probation officers may communicate and coordinate with treatment providers to refer for services or seek clinical guidance. For example, one participant explained: “We reach out to the provider and try to establish a form of communication so that we know what they have going on, and again, so that we can hopefully get guidance as to the most effective way to get them successfully through their probation.”

Other forms of coordination and collaboration include establishing close networks with treatment providers to streamline and speed up access to services. One participant explained:

I try to network, meeting programs, talking to programs. My officers have a whole bunch of agencies that they coordinate with, contact with, work very closely with to assist. As a supervisor, too, I try to continue to build my network of different programs, of trying to streamline the process, of hoping to get clients into programs at a more expedited rate based upon situations. Just having a name or a contact and explaining the situation has done wonders. It's all about networking and trying to get it out there of what we do and what we're trying to accomplish.

Another way to streamline services is to create formalized agreements with treatment providers. One participant indicated that they have providers go through a RFP process to ensure those they contract with meet certain criteria, including being licensed and using best practices.

#### Sanctions and modifications

Across the counties reporting details about their mental health caseloads, the majority (67%, *n* = 53) reported not having greater flexibility to modify sanctions for people with mental illnesses on probation. Similarly, 80% (*n* = 39) of counties with mental health caseloads reported not having enhanced flexibility to seek modifications to probation conditions. There were no statistically significant differences in the flexibility to modify sanctions or probation conditions between rural and urban counties’ select mental health probation approaches.

### Additional supervision strategies beyond mental health caseloads

This section describes additional strategies that agencies use to supervise people with mental illnesses. Strategies ranged from embedding mental health treatment within the agency to modifying the probation strategies used for the general probation population to meet the needs of people with mental illnesses. Mental health caseloads and the strategies that follow are not mutually exclusive. Rather, mental health probation officers may integrate a number of these strategies into their role; however, they are described separately because they can and are implemented outside the context of a mental health caseload.

#### Training for standard officers

Given the large numbers of people on probation with mental illnesses, the vast majority will be supervised by standard officers (i.e., not specialty mental health probation officers). Consequently, some agencies have implemented mental health training for standard officers as well. Across the 179 counties represented in the survey, 36% (*n* = 64) required that standard officers receive mental health training. Of those counties requiring mental health training for standard officers, the average number of training hours was 8 (SD = 7.06) and officers were required to repeat the training annually in 63% (*n* = 40) of respondent counties. CIT training was required in 8% (*n* = 15) of respondent counties, mental health first aid was required in 28% (*n* = 51), and another type of mental health crisis de-escalation training was required for standard officers in 47% (*n* = 84) of respondent counties.

#### Embedding services within probation setting

Another supervision strategy identified by survey respondents and interview participants was embedding or co-locating treatment or mental health care and coordination within the probation setting. Survey respondents described embedded treatment as “mental health provided withing the community corrections agency,” “embedded therapists,” “severe mental health designated clinician,” “psychiatric social workers on staff to conduct assessments,” and “behavioral health staff embedded within probation staff.” Another survey respondent described the embedded strategy as “mental health specific cognitive behavioral intervention curriculum delivered at Day Reporting Center.” One participant indicated that because referrals had long backlogs, they used federal grant money to do their own testing. Another agency said:

We have a social worker assigned to that unit that then will do psychosocial [assessments] and then if she determines that the person needs further evaluation, we have two consulting psychologists that we can call in to do any testing… others will be referred out to mental health agencies for reports and then the probation officers will get copies of those.

#### De Facto mental health caseloads

Some probation officers serve as informal experts or consultants or are the “go-to” officer to whom mental health cases are assigned. Often, these are officers who are more skilled, experienced, or interested in working with individuals with mental illnesses. This approach was sometimes used by agencies that did not have the resources or numbers of staff to create a designated mental health caseload, for example: “We have a couple agents that are very much in tune to the mental health needs, and so I’ve allowed them to take the lead on that. They’re an office resource for agents even at night. Sometimes agents will call and say, ‘Hey, I have a client struggling, what do you think is available?’” Another participant explained, “We used to have a dedicated mental health caseload. The numbers aren’t high enough now to justify it. And of course, staffing levels are kind of constantly an issue. So, now they’re just kind of divvied up to whoever gets them. We have some more senior POs that we’ll assign the more troublesome guys to just because they’re more experienced.”

#### Case staffing

Probation case staffing entails consultations of probation officers with other probation agency staff, including other officers and/or supervisors. One interviewee described the value of having experienced probation officers in case staffing:

One thing we do well is staff cases with each other, and be like, "Hey, I've got this [person] and he seemed off last time he was here. Next time he comes in, can you sit in with me, or can you keep an ear open and see what you think when I talk to him?" There’s a lot of staffing going on between officers that they’re not real sure how to handle the case, especially with new officers.”

In addition to staffing cases with other probation officers and supervisors, some agencies do multidisciplinary case staffing. Multidisciplinary case staffing entails probation agency staff meeting with non-probation agency staff (i.e., multiple disciplines) – often, some combination of behavioral health treatment providers, other social service providers, lawyers, and law enforcement. One participant explained:

We work with the lead mental health agency in the state, the [State’s Department of Behavioral Health Services], and they provide case management and other things… They’ll discuss what they’re hearing from the client or from family members, and they’ll be able to have a conversation with the clinician who will be able to help them make a decision if there’s decompensation occurring, what needs to be done.

#### Using a problem-solving approach and cognitive behavioral interventions

Probation officers employ the use of problem-solving strategies during supervision (e.g., working with the individual to overcome barriers to compliance). This problem-solving orientation allows for more flexible, solution-oriented, personalized supervision. One participant summarized multiple aspects of a problem-solving approach as follows:

I am in contact with different treatment providers multiple times a day to keep tabs on individuals. I know if someone doesn't show up for a group counseling session; I get a call right after that group to let me know, that way I can reach out and make sure things are okay… I have a department cell phone and everyone on my caseload knows if there are any concerns at all, or questions, or you need help with anything, they can text my number at any time. With just increased help with things, I have someone who is coming in and they are frustrated, "I don’t have a car. I don't have my license. So, what do we need to do?" I take those extra steps with them to make sure they are set up for success and don’t become overwhelmed.”

Relatedly, officers also use cognitive behavioral interventions – that is, identifying and altering negative patterns of thinking—as part of their problem-solving approach. As one participant explained:

We have a program called Cognitive Behavioral Intervention. It’s CBI… it’s a lengthy program … the participants in the program do workbooks and classes and present each step, and the classes all go around how to make different, better decisions when you’re faced with a challenge. Some of that is around mental health side. Some of it’s not. Some of it’s just, “I’ve done it this way,” or, “I don’t know any other way to do it, and that’s how I’m going to do it.”

## Discussion

This article describes approaches to supervising people with mental illnesses on probation in specialized or general caseloads, including screening and identification of mental illnesses, and compares results across rural and urban counties. Strategies for identifying mental illnesses varied, with most agencies using risk needs assessments (RNAs), self-report items asked during the intake process, or information from pre-sentencing reports. Less than a third of respondents reported using screening and assessment tools specific to mental health or having a system that tracks or “flags” mental illnesses. The lack of mental health flags in agencies’ records management systems, coupled with the lack of mental health screening and assessment questions, likely leads to under- or misidentification of people with mental illnesses on probation, and the agency then estimates the prevalence rates (rather than agency data).

Despite the potential impact that limited mental health screening could have on calculating prevalence rates, results of this study showed that, regardless of the source of the reported prevalence rate (i.e., respondent estimate, agency data, other), prevalence rates fell within the estimates from empirical research studies, which range from 16 to 27% (e.g., Crilly et al., 2009; Ditton, 1999). These prevalence rates were also consistent across rural and urban counties; however, the availability of supervision strategies for addressing mental illness was not. Specifically, compared to urban counties, rural counties were less likely to report that they had specialized mental health caseloads. This leaves people on probation in rural areas with fewer interventions aimed to reduce recidivism among people with mental illnesses.

Results also showed wide variation in mental health training requirements for probation officers. This variation and lack of standardization of training means that officer capacity and knowledge base also vary. Although there is no existing empirical research to demonstrate wide variation in mental health knowledge and skills across officers, it is reasonable to assume that significant differences in mental health training result in significant differences in officer capacity to supervise people with mental illnesses.

Lastly, results from the survey and follow-up interviews showed wide variation in the types of strategies used for supervising people with mental illnesses. Some agencies implement mental health caseloads or embed mental health services within probation while other agencies may implement a one-time mental health training for all probation officers. The wide variation in implementation of supervision strategies presents (1) an opportunity for counties to select from a variety of strategies and tailor them to fit the needs of their local context and (2) a challenge of building the evidence base for a single strategy or set of strategies (e.g., the prototypical model advanced by Skeem et al., 2006).

### Limitations

There are several factors that should be considered when interpreting the study findings. First, plans for this analysis began in 2019, prior to the onset of the COVID-19 pandemic. In March 2020, the timeline of the study and the aims were modified due to the impact COVID-19 was having on corrections systems across the country. The research team delayed the initiation of data collection by several months to avoid interrupting the work of corrections agencies at a critical time in their efforts to curb the spread of COVID-19 among their populations. However, given the differences in impact of COVID-19 on correctional institutions across the country, initiation of the study inevitably occurred when agencies were under strain. Although this study had a strong response rate of over 50%, the research team believes that the COVID-19 pandemic had a significant impact on the recruitment process and study timeline.

A second factor that impacted the study design and results is the governance and administration of probation agencies. Governance and administration of probation departments vary greatly by state. These vastly different administrative structures appear to fall within the following levels of governance: (1) state, (2) district, (3) regional or jurisdiction level, (4) county, or (5) municipal. Additionally, supervision may be privately operated (e.g., via contract with private companies) or operated by the government. Thus, one state may have state-level governance but county-level or regional administration; in other states, governance and administration may both fall under the counties’ purview. While variation in governance and administration helps explain some variations in approaches, it also poses significant challenges for obtaining county-level information. For instance, state-run agencies may not disaggregate their data by county, while other agencies may base their answers on the region or circuit in which the county is located, making it difficult to interpret the results (Kaeble, 2023).

Lastly, results pertaining to mental health caseloads are not generalizable. These findings are based on data from counties that have mental health caseloads and subsequently provided additional details about those caseloads. Consequently, it is possible that some respondent counties have mental health caseloads but did not provide additional details. Given that the aim of this aspect of the study was to describe and not to generalize, the impact of this limitation on study findings and key takeaways is negligible.

### Implications

Results from this study support promoting the capacity of probation agencies to identify and supervise people with mental illnesses. Specifically, we suggest four areas of practice, policy, and research: (1) accept model variation while promoting responsivity and rehabilitative approaches; (2) grow the research on strategies for supervising people with mental illnesses; (3) promote standardization of mental health screening processes; and (4) enhance mental health training for mental health probation officers and standard probation officers.

#### Accept model variation while promoting responsivity and rehabilitative approaches

Although developing evidence-based practices (EBPs) through rigorous research methods is a primary goal in the field, adherence to EBPs requires fidelity and uniformity that may not be feasible or advisable in some jurisdictions. Variation in implementation should not necessarily be viewed as a lack of fidelity to a single model of specialty mental health probation; rather, these differences manifest for a number of reasons that demand tailoring programs to the local context. For instance, differences in resources available in rural and urban settings means that programs and interventions that may have been tested and subsequently demonstrated efficacy in an urban environment may not be effective when generalized to a rural environment.

Additionally, probation is implemented within a larger state, regional, and local sociopolitical context. Specifically, given that governance and administration of probation is decentralized, operations and programming are designed and implemented by the administrative body (e.g., state, circuit, county) which is influenced by the jurisdiction’s political and philosophical orientation, including how the agency operationalizes probation’s retributive and/or rehabilitative role. These political and philosophical differences will create variation in programming and implementation, focusing not on *whether* a rehabilitative approach is implemented for people with serious mental illnesses, but *how* it is implemented. In short, regardless of political and philosophical differences across jurisdictions, the complex challenges that people with serious mental illnesses face, and the barriers to supervision compliance that these challenges create, a rehabilitative orientation to supervising people with mental illnesses should be mandated.

#### Grow the research on individual model components or strategies

Although it is important to continue promoting rigorous research on the prototypical specialty mental health probation model (Skeem & Louden, 2006), it is also important to promote rigorous research on the efficacy of specific program components in improving supervision outcomes for people on probation with mental illnesses. Although implementing a package of supervision strategies (e.g., the prototypical mental health probation model) is feasible and desirable in some jurisdictions, it may not be in others. Consequently, more attention could be paid to research on the impact of different types of supervision strategies, both as standalone strategies and as part of a larger approach. Using more rigorous research methods to isolate and measure the impact of discrete supervision strategies is the logical next step for the field. This focus should include supervision strategies beyond mental health caseloads. For example, research can focus on embedded mental health treatment within probation, case staffings, and other techniques that are used with the general population.

Additionally, research on specific strategies for supervising people with mental illnesses should include a dual aim focused on implementation science. Implementation science refers to the study of research methods focused on enhancing the implementation or uptake of evidence-based practices into real world settings (Bauer & Kirchner, 2020; Bauer et al., 2015). Within the context of mental health probation, in addition to learning whether a given supervision strategy was effective, it is important to understand how it was implemented, whether it was implemented with fidelity, the characteristics of the context in which it was implemented, factors that impacted implementation, and the effectiveness of efforts aimed at addressing barriers to implementation (i.e., implementation strategies). Implementation science methods are widely used in health services and are increasingly applied in criminal justice settings via initiatives funded by the National Institute of Drug Abuse (e.g., Criminal Justice Drug Abuse Treatment Studies, Justice Community Opioid Innovation Network, Juvenile Justice – Translational Research on Interventions for Adolescents in the Legal System; Ducharme et al., 2021; Knight et al., 2015; National Institutes of Health [NIH], 2002; NIH, 2007); however, with few exceptions (e.g., Van Deinse et al, 2019, 2021, 2022), there has been scant application of implementation science methods to mental health probation.

Research on the effectiveness of strategies for supervising people with mental illnesses, coupled with findings from implementation science research, could inform the development of a mental health probation toolkit. Such a toolkit could be an important resource for agencies to use in developing or adjusting mental health probation programs to match the needs and resources of their jurisdiction. For example, a toolkit could describe a number of strategies for supervising people with mental illnesses, the strength of the evidence base for each strategy, specific information about key factors that enhance or inhibit implementation of the specific strategy, and successful efforts to address barriers to implementing these supervision strategies. Although information and examples about best practices in supervision exist, actionable implementation-focused information is notably absent.

#### Promote standardization of mental health screening processes

Mental health screening is the starting point for addressing an individual’s mental health needs. For instance, a positive indication on a mental health screening instrument may be used as a signal for further mental health assessment and subsequent treatment referral and engagement. Due to probation agencies’ low rates of screening for mental illnesses, coupled with the use of instruments that may have insufficient or unknown psychometric properties for screening for mental illnesses, individuals in need of services and supports may not be adequately identified.

Research indicates that, while mental illness alone is not a direct predictor of criminal justice involvement, it is a criminogenic need that can destabilize a person and compound criminogenic risk factors (e.g., substance use) that lead to further involvement with the criminal legal system (Bonta et al., 2014; Prins et al., 2015). For example, many justice-involved individuals with mental illnesses also have co-occurring substance use disorders, which can exacerbate poor mental health outcomes, increase treatment costs, and negatively impact housing, recidivism, and violence (Van Dorn et al., 2017). Most agencies in this study used some type of screening or assessment tool as part of their intake process; however, few used standardized mental health screening tools and relied on information from risk need assessments, which may not have a validated mental health screening embedded in the tool or may not contain mental health related items. Agencies should consider using standardized mental health-specific screening instruments with sufficient reliability and validity to identify people with potential mental health concerns which can help inform agency decision making around resource allotment by better understanding the scope of mental health needs among the population. This is particularly important for any agency that is considering implementing mental health probation strategies. A screening instrument can be used to help make decisions about eligibility criteria and referral for further mental health assessments.

When selecting mental health screening instruments, agencies should consider their own resource capacity, including what types of training a person would need in order to administer the mental health screening instrument. For example, some agencies reported using the Brief Jail Mental Health Screen (Steadman et al., 2005). This tool is widely used in jail settings and does not require enhanced training and skills in mental health. The BJMHS is just one example of many screening instruments and agencies considering adopting a screening instrument should consider how they plan to use the screening tool and any known limitations associated with the instrument (e.g., gender bias, racial bias, high cost, poor reliability and valididty).

#### Enhance mental health training for standard and specialty officers

It is critically important for probation agencies to assess the degree to which their existing mental health training protocol meets the needs of the officers to competently supervise people with mental illnesses. This is true for standard caseloads as well as mental health caseloads. Given that a majority of people with mental illnesses on probation do not have access to specialty mental health probation approaches, they are likely placed with standard probation officers who often do not have mental health training beyond what may have been offered during basic training when they were onboarded. Consequently, advanced training in mental illness is equally important for standard officers as it is specialty mental health probation officers. Establishing best practice standards for mental health training for probation officers could include specification of content tailored to the type of role the officer is in (e.g., standard officer vs. mental health probation officer), requirements for length and format for the training, and frequency (e.g., booster sessions, annual training), and specification regarding mandated versus voluntary training. Additionally, more research needs to be done to provide evidence of different training modalities’ efficacy in improving supervision outcomes. For examples, it is not clear whether mental health training programs aimed at a more general audience, such as Mental Health First Aid, provide officers with the practical information needed to improve their supervision of people with mental illnesses. Rather, research should explore the effectiveness of training curriculum specific to supervising people with serious mental illnesses who are involved in the criminal legal system.

## Conclusion

Despite the large numbers of people with mental illnesses on probation, a majority of probation agencies that participated in this study did not have formal mechanisms for identifying people with mental illnesses on their caseloads or mental health supervision strategies. In the absence of screening and identification methods and supervision strategies, probation agencies have limited capacity to supervise people with mental illnesses. This limitation is significant given that individuals with mental illnesses have elevated risk of poor criminal justice outcomes. Probation agencies should adopt a rehabilitative orientation to supervising people with mental illnesses beginning with developing a mechanism for identifying people with potential mental health needs and creating a protocol for mental health screening and referral for treatment. Additionally, probation agencies should enhance mental health training for both specialized and standard officers and develop a set of supervision strategies for working with people with mental illnesses.

## Data Availability

Data analyzed in this study may be made available from the corresponding author upon reasonable request.
